# Molecular Characterization of Branchial *aquaporin 1aa* and Effects of Seawater Acclimation, Emersion or Ammonia Exposure on Its mRNA Expression in the Gills, Gut, Kidney and Skin of the Freshwater Climbing Perch, *Anabas testudineus*

**DOI:** 10.1371/journal.pone.0061163

**Published:** 2013-04-09

**Authors:** Yuen K. Ip, Melody M. L. Soh, Xiu L. Chen, Jasmine L. Y. Ong, You R. Chng, Biyun Ching, Wai P. Wong, Siew H. Lam, Shit F. Chew

**Affiliations:** 1 Department of Biological Science, National University of Singapore, Kent Ridge, Singapore, Republic of Singapore; 2 Natural Sciences and Science Education, National Institute of Education, Nanyang Technological University, Singapore, Republic of Singapore; 3 NUS Environmental Research Institute, National University of Singapore, Kent Ridge, Singapore, Republic of Singapore; University of Sydney, Australia

## Abstract

We obtained a full cDNA coding sequence of *aquaporin 1aa* (*aqp1aa*) from the gills of the freshwater climbing perch, *Anabas testudineus*, which had the highest expression in the gills and skin, suggesting an important role of Aqp1aa in these organs. Since seawater acclimation had no significant effects on the branchial and intestinal *aqp1aa* mRNA expression, and since the mRNA expression of *aqp1aa* in the gut was extremely low, it can be deduced that Aqp1aa, despite being a water channel, did not play a significant osmoregulatory role in *A. testudineus*. However, terrestrial exposure led to significant increases in the mRNA expression of *aqp1aa* in the gills and skin of *A. testudineus*. Since terrestrial exposure would lead to evaporative water loss, these results further support the proposition that Aqp1aa did not function predominantly for the permeation of water through the gills and skin. Rather, increased *aqp1aa* mRNA expression might be necessary to facilitate increased ammonia excretion during emersion, because *A. testudineus* is known to utilize amino acids as energy sources for locomotor activity with increased ammonia production on land. Furthermore, ammonia exposure resulted in significant decreases in mRNA expression of *aqp1aa* in the gills and skin of *A. testudineus*, presumably to reduce ammonia influx during ammonia loading. This corroborates previous reports on AQP1 being able to facilitate ammonia permeation. However, a molecular characterization of Aqp1aa from *A. testudineus* revealed that its intrinsic aquapore might not facilitate NH_3_ transport. Hence, ammonia probably permeated the central fifth pore of the Aqp1aa tetramer as suggested previously. Taken together, our results indicate that Aqp1aa might have a greater physiological role in ammonia excretion than in osmoregulation in *A. testudineus*.

## Introduction

Aquaporins (AQPs) are an extended family of integral membrane proteins that mediate facilitated transmembrane water transport [1]. They exist as tetramers [2] with each monomer possessing its own functional channel [3], [4]. At the center of the four monomers lies a fifth pore composed mainly of hydrophobic amino acids. In mammals, 13 homologs of AQPs (0–12) are known, with some also involved in the exchange of glycerol and other low molecular weight solutes such as urea, CO_2_, or NH_3_ [5], [6].

The most ubiquitous and extensively studied AQP is aquaporin 1 (AQP1), which was the first AQP discovered while Preston and Agre [7] were identifying Rh blood group polypeptides in the erythrocyte plasma membrane. Human AQP1 has 269 amino acids of 28 kDa, and contains 6 transmembrane regions with 5 connecting loops, of which 3 (A, C, and E) are located outside the cell and 2 (B and D) within the cytoplasm [8]. Two identical asparagine–proline–alanine motifs at residues 76–78 (in cytoplasmic loop B) and 192–194 (in extracellular loop E) are connected to each other within the membrane, forming a single narrow aqueous pathway (aquapore) of 2.8 Å in diameter at the narrowest point (the constriction region) as calculated by electron crystallography [9], [10], [11]. The outer constriction region contains an aromatic/arginine motif, which acts as a selective filter [12]. In some AQPs, the polarity and diameter of this constriction region is tuned to facilitate the transport of polar solutes other than water [13], [14].

Since H_2_O and NH_3_ have similar molecular sizes and charge distribution, several studies examined the role of aquaporins, in particular AQP1, AQP3, AQP8, and AQP9, in transmembrane NH_3_ transport. Nakhoul et al. [15] expressed human AQP1 in *Xenopus* oocytes, which have low NH_3_ permeability, and concluded that NH_3_ permeability was enhanced by AQP1. However, not all studies have confirmed that AQP1 can transport NH_3_ [16], [17]. Holm et al. [16] expressed human aquaporins AQP8, AQP9, AQP3, and AQP1 in *Xenopus* oocytes to study the transport of NH_3_ and NH_4_^+^ under open-circuit and voltage-clamped conditions, and concluded that apart from being water channels, AQP3, AQP8 and AQP9 also supported significant fluxes of NH_3_ and NH_4_^+^. Yet, based on a similar technique, Musa-Aziz et al. [18] reported recently that human AQP1 enhanced NH_3_ influx significantly more than AQP4 and AQP5 in *Xenopus* oocytes, pointing to facilitated transport of NH_3_ by AQP1 and contradicting the report of Holm et al. [16] that AQP1 did not significantly affect NH_3_ transport.

Homologs of *aqp1* have been identified in several species of teleost fish [18], [19], including the European eel (*Anguilla anguilla*) [20], [21], Japanese eel (*Anguilla japonica*) [22], [23], gilthead seabream (*Sparus aurata*) [24], [25], [26], sole (*Solea senegalensis*) [27], zebrafish (*Danio rerio*) [27], black seabass (*Centropristis striata*) [28], silver seabream (*Sparus sarba*) [29], [30], European seabass (*Dicentrarchus labrax*) [31], black porgy (*Acanthopagrus schlegeli*) [32], killifish (*Fundulus heteroclitus*) [27], rainbow wrasse (*Coris julis*) [33], and Indian catfish (*Heteropneustes fossilis*) [34]. There are indications that Aqp1aa/Aqp1ab could be involved in osmoregulation in gills, gut and possibly kidneys of teleosts during salinity acclimation. Apical Aqp1aa may function in collaboration with basolateral Aqp3 in transepithelial water transport and prevention of cell swelling in the gills of some freshwater fishes [26], [30]. Aqp1aa/Aqp1ab may also be involved in the absorption of water in the gut of marine fish [22], [24], [31], [35]. However, there is a dearth of knowledge on the possible roles of Aqp1aa/Aqp1ab in water balance and/or ammonia transport in fish during emersion or exposure to environmental ammonia, especially in those amphibious air-breathing species with high ammonia tolerance.

The climbing perch, *Anabas testudineus* (Bloch), is a freshwater teleost belonging to Order Perciformes and Family Anabantidae. It can be found in canals, lakes, ponds, swamps and estuaries in tropical Asia, and can tolerate extremely unfavorable water conditions [36]. It possesses accessory breathing organs (or labyrinth organs) in the upper part of the gill-chambers, which facilitate the utilization of atmospheric air [37], [38], [39]. Periodically, it approaches the water surface to gulp air, which is channeled to the accessory breathing organs for gaseous exchange. During drought, *A. testudineus* stays in pools associated with submerged woods and shrubs [40], or buries under the mud [41]. To search for a new habitat, it can travel long distances on land between pools of water, covering several hundred metres per trip when the air is sufficiently humid [42]. During emersion, *A. testudineus* can maintain relatively low plasma urea and ammonia concentrations due to its ability to actively excrete ammonia through the gills and skin [43]. Since it is capable of active ammonia excretion, it also exhibits extraordinarily high tolerance of environmental ammonia (∼100 mmol l^−1^ NH_4_Cl at pH 7.0). In addition, *A. testudineus* can acclimate from freshwater to seawater through a progressive increase in salinity [44]. Recently, it has been demonstrated that active extrusion of Na^+^ during seawater acclimation and active excretion of NH_4_^+^ during exposure to environmental ammonia in freshwater in the gills of *A. testudineus* involve similar transport mechanisms, Na^+^/K^+^-ATPase, Na^+^:K^+^:2Cl^−^ cotransporter and cystic fibrosis transmembrane conductance regulator, but different types of mitochondrion-rich cells [45], [46], [47]. NH_4_^+^ can be transported, in substitution of K^+^, from plasma into mitochondrion-rich cells through the basolateral Na^+^:K^+^:2Cl^−^ cotransporter [45], and exit the apical membrane through an unknown NH_4_^+^ transporter down a favorable electrochemical potential generated by the excretion of Cl^−^ and/or HCO_3_^−^ through the apical cystic fibrosis transmembrane conductance regulator [47]. The main function of Na^+^/K^+^-ATPase in active NH_4_^+^ excretion is to maintain intracellular Na^+^ and K^+^ homeostasis, instead of transporting NH_4_^+^ directly into mitochondrion-rich cells [46]. Since Aqp1aa is exclusively localized in the branchial epithelium of gilthead seabream [48], and Aqp1-like water channels are found in mitochondrion-rich cells in the gills of rainbow wrasse [33], the first objective of this study was to obtain the full cDNA sequence of *aqp1aa* from the gills of *A. testudineus*. The second objective was to examine the tissue expression of *aqp1aa* in *A. testudineus*. The third objective was to determine the mRNA expression of *aqp1aa* in the gills, anterior gut, posterior gut, kidney and skin of *A. testudineus* kept in freshwater (control) or exposed to seawater (salinity 30; 1 or 6 days), terrestrial conditions (1 day), or environmental ammonia (100 mmol l^−1^ NH_4_Cl; 1 day) using quantitative real-time PCR (qPCR). The hypothesis tested was that *aqp1aa*/Aqp1aa might have a more prominent role in ammonia excretion than in osmoregulation in *A. testudineus* which, despite being regarded commonly as a freshwater teleost, could acclimate to seawater, survive terrestrial exposure and tolerate high concentrations of environmental ammonia.

## Materials and Methods

### Animals

Specimens of *A. testudineus* (25–45 g body mass) were purchased from a local fish distributor. Fish were kept in dechlorinated tap water (freshwater; pH 6.8–7.0) at 25°C in fiberglass tanks with a continuous flow through system for at least 2 weeks under a 12 h light: 12 h dark regime before experiments. No aeration was provided because *A. testudineus* is an obligatory air-breather. They were fed frozen blood worms once every two days. Procedures adopted in this study were approved by the Institutional Animal Care and Use Committee of the National University of Singapore (IACUC 021/10 and 098/10).

### Experimental conditions and collection of samples

Control fish (*N* = 6) were immersed in 25 volumes (v/w) of freshwater. For fish exposed progressively to seawater, they (*N* = 12) were randomly selected and transferred to fiberglass tanks containing freshwater (pH 7.0) on day 0 and subsequently, to salinity 10 (pH 7.4) on day 1, salinity 15 (pH 7.6) on day 2, salinity 20 (pH 7.8) on day 3, salinity 25 (pH 8.1) on day 4, and salinity 30 (seawater, pH 8.3) on day 5. Some fish were kept in seawater for an additional 6 days. Natural seawater was collected from the sea at least 1 km away from the coast of the Singapore main island. Waters of different salinities were prepared by mixing seawater with an appropriate quantity of freshwater. Salinity was monitored using a YSI Model 30/10 FT salinometer (Yellow Springs Instrument Co. Inc, Ohio, USA). Fish (*N* = 6) were killed for sample collection after 1 or 6 days in seawater. During salinity acclimation, fish were fed frozen blood worms on alternate days but fasted 2 days before sample collection. For fish exposed to terrestrial conditions, they (*N* = 6) were randomly selected, fasted for 2 days and transferred to fiberglass tanks containing a thin film of freshwater (10 ml) for one day. Another batch of fish (*N* = 6) were randomly selected and exposed to 100 mmol l^−1^ NH_4_Cl at pH 7.0 for one day. Fish were anaesthetized with 0.05% neutralized MS-222 and killed with a strong blow to the head. Gills, gut, kidney, skin, brain and accessory breathing organs were quickly excised, cooled in liquid N_2_ and stored at −80°C.

### Total RNA extraction and cDNA synthesis

The total RNA of the gill sample was extracted using the chaotropic extraction protocol of Whitehead and Crawford [49], and further purified using the Qiagen RNeasy Mini Kit (Qiagen GmbH, Hilden, Germany). Following isolation, RNA was quantified spectrophotometrically using a Hellma traycell (Hellma GmbH & Co. KG, Müllheim, Germany). The RNA quality was checked electrophoretically to verify RNA integrity and RNA was stored at −80°C. First strand cDNA was synthesized from 1 µg of total RNA using oligo(dT)_18_ primer and the RevertAid™ first strand cDNA synthesis kit (Fermentas International Inc., Burlington, ON, Canada).

### Polymerase Chain Reaction (PCR)

The partial *aqp1aa* sequence was obtained using primers (Forward: 5′-ASATMAGYGGHKCCCA-3′; Reverse: 5′-CCAGTAHACCCARTG-3′) designed from the highly conserved regions from multiple alignments of the *aqp1* sequences from various fish species available in Genbank (http://www.ncbi.nlm.nih.gov/Genbank/). Polymerase chain reaction (PCR) was performed in Biorad Peltier thermal cycler (Biorad, Hercules, CA, USA) using Dreamtaq polymerase (Fermentas International Inc.). The cycling conditions were 95°C for 3 min, followed by 35 cycles of 95°C for 30 s, 55°C for 30 s, 72°C for 2 min and a final extension of 72°C for 10 min. PCR products were separated by electrophoresis in 1% agarose gel. Bands of the estimated *aqp1aa* sizes were excised and purified from the gel using QIAquick® Gel Extraction Kit (Qiagen GmbH) according to manufacturer's protocol. Purified PCR products were subjected to cycle sequencing using BigDye® Terminator v3.1 Cycle Sequencing Kit (Applied Biosystems, Foster City, CA, USA) and sequenced using the 3130XL Genetic Analyzer (Applied Biosystems).

### Rapid amplification of cDNA ends (RACE)-PCR

Total RNA (1 µg) isolated from the gills of *A. testudineus* in freshwater was reverse transcribed into 5′-RACE-Ready cDNA and 3′RACE-Ready cDNA using SMARTer™ RACE cDNA Amplification kit (Clontech Laboratories, Mountain View, CA, USA). RACE-PCR was performed using the Advantage® 2 PCR kit (Clontech Laboratories) to generate the 5′ and 3′ cDNA fragments, with 5′-GGCTTAACGCTCTCAGTGGTGTTACCC-3′ and 5′-GTAACACCACTGAGAGCGTTAAGC-3′, respectively. RACE-PCR cycling conditions were 25 cycles of 94°C for 30 s, 65°C for 30 s and 72°C for 4 min. RACE-PCR products were separated using gel electrophoresis, purified and sequenced.

The partial fragments of *aqp1aa* obtained from the gills of *A. testudineus* were aligned using BioEdit [50] to obtain the full-length nucleotide coding sequence, which were then translated into amino acid sequence. The deduced amino acid sequence was aligned and compared with selected Aqp from various animal species using BioEdit. The sequence identity generated was used to confirm the identity of the Aqp1aa from *A. testudineus*. Transmembrane domains were identified using the MEMSATS & MEMSAT-SVA provided by PSIPRED protein structure prediction server (http://bioinf.cs.ucl.ac.uk/psipred/) [51].

### Phylogenetic analysis

Amino acid sequences of Aqp1 from other animals were obtained from Genbank or UniProtKB/TrEMBL with the following accession numbers: *Acanthopagrus schlegelii* Aqp1 (ABO38816.1), *Anguilla anguilla* Aqp1 (CAD92028.1), *Anguilla anguilla* Aqp1b (ABM26906.1), *Anguilla japonica* Aqp1 (BAC82109.1), *Anguilla japonica* Aqp1b (BAK53383.1), *Cynoglossus semilaevis* Aqp1 (ADG21868.1), *Dicentrarchus labrax* Aqp1 (ABI95464.2), *Diplodus sargus* Aqp1 (AEU08496.1), *Fundulus heteroclitus* Aqp1 (ACI49538.1), *Heteropneustes fossilis* Aqp1b (ADK87346.1), *Homo sapiens* AQP1 (CAQ51480.2), *Hyla japonica* AQP-h1 (BAC07470.1), *Mus musculus* AQP1 (EDK98728.1), *Protopterus annectens* Aqp1 (BAI48049.1), *Rattus norvegicus* AQP1 (NP_036910.1), *Rhabdosargus sarba* Aqp1 (AEG78286.1), *Salmo salar* Aqp1 (NP_001133472.1), *Sparus aurata* Aqp1a (ABM26907.1), *Sparus aurata* Aqp1b (ABM26908.1), *Takifugu obscurus* Aqp1 (ADG86337.1), *Xenopus laevis* AQP1 (NP_001085391.1), *Xenopus tropicalis* AQP1 (NP_001005829.1) and *Anopheles gambiae* Aqp1 (BAI60044.1) as an outgroup. These sequences were aligned using ClustalX2 and phylogenetic analysis was performed using neighbor-joining method and 100 bootstrap replicates with Phylip [52].

### Tissue expression

Total RNA (1 µg) isolated from gills, anterior gut, posterior gut, kidney, skin, brain and accessory breathing organs of *A. testudineus* kept in freshwater were reverse transcribed into cDNA using oligo(dT)_18_ primer and the RevertAid™ first strand cDNA synthesis kit (Fermentas International Inc.). PCR was performed on the cDNAs of these tissues using forward primer 5′-AATTCAAGAGCAAGAACTTCTG-3′ and reverse primer 5′-GAGCGACACCTTCACCTC-3′ to detect the mRNA expression of each gene in various tissues. Each PCR was carried out in 10 µl reaction volumes using Dreamtaq polymerase (Fermentas International Inc.) with thermal cycling conditions: 95°C for 3 min, followed by 30 cycles of 95°C for 30 s, 55°C for 30 s, 72°C for 30 s and a final extension of 72°C for 10 min. PCR products were then separated by electrophoresis in 2% agarose gel.

### qPCR

RNA from gill samples were treated with Deoxyribonuclease I (Sigma-Aldrich Co., St. Louis, MO, USA), to remove any contaminating genomic DNA. First strand cDNA was then synthesized from 1 µg of total RNA using random hexamer primer and the RevertAid™ first strand cDNA synthesis kit (Fermentas International Inc.).

qPCR was performed in triplicates using a StepOnePlus™ Real-Time PCR System (Applied Biosystems). The standard cDNA (template) was serially diluted in 1X TE buffer (1 mmol^−1^ Tris, 0.1 mmol l^−1^ EDTA, pH 8.0) (from 10^6^ to 10^2^ specific copies/2 µl). The qPCR reactions contained 5 µl of 2X Fast SYBR® Green Master Mix (Applied Biosystems), 0.3 µmol l^−1^ of forward (5′-AATTCAAGAGCAAGAACTTCTG-3′) or reverse primers (5′-GAGCGACACCTTCACCTC-3′), and cDNA (equivalent to 1 ng of RNA) or standard (2 µl) in a total volume of 10 µl. Cycling conditions were 95°C for 20 s (1 cycle), followed by 45 cycles of 95°C for 3 s and 60°C for 30 s. Data (threshold cycle as C_T_ values) were collected at each elongation step. Runs were followed by melt curve analysis by increasing from 60°C to 95°C in 0.3°C increments to confirm the presence of only a single product. The PCR products were separated in a 2% agarose gel to verify the presence of a single band.

In order to determine the absolute quantity of *aqp1aa* transcripts in a qPCR reaction, efforts were made to produce a pure amplicon (standard) of a defined region of *aqp1aa* cDNA from the gills of *A. testudineus* following the methods of Gerwick et al. [53]. PCR was performed with *aqp1aa* qPCR primers and cDNA as a template in a final volume of 25 µl with the following cycling conditions: initial denaturation of 95°C for 3 min, followed by 35 cycles of 95°C for 30 s, 60°C for 30 s and 72°C for 30 s and 1 cycle of final extension of 72°C for 10 min. The PCR product was separated in a 2% agarose gel. The product was excised and purified using QIAquick gel extraction kit (Qiagen GmbH). The *aqp1aa* fragment in the purified product was cloned using pGEM®-T Easy vector (Promega Corporation, Madison, WI, USA). The presence of the insert in the recombinant clones was confirmed by sequencing. The cloned circular plasmid was quantified using a spectrophotometer. A standard curve was obtained from plotting threshold cycle (C_T_) on the *Y* axis and the natural log of concentration (copies/µl) on the *X* axis. The C_T_, slope, PCR efficiency, *Y* intercept and correlation coefficient (*R* ^2^) were calculated using the default setting of StepOne™ Software v2.1 (Applied Biosystems). Diluted standards were stored at −20°C. The PCR efficiency for *aqp1aa* was 96.9%. The quantity of transcript in an unknown sample was determined from the linear regression line derived from the standard curve and expressed as copies of transcripts per ng cDNA.

### Statistical analysis

Results were presented as means ± standard errors of the mean (S.E.M.). Independent two-tailed *t* test or one-way analysis of variance (ANOVA), followed by multiple comparisons of means by the Tukey test, were used in the evaluation of the differences between means where applicable. Differences were regarded as statistically significant at *P*<0.05.

## Results

### Nucleotide sequence, translated amino acid sequence and phylogenetic analysis

**Figure 1:**
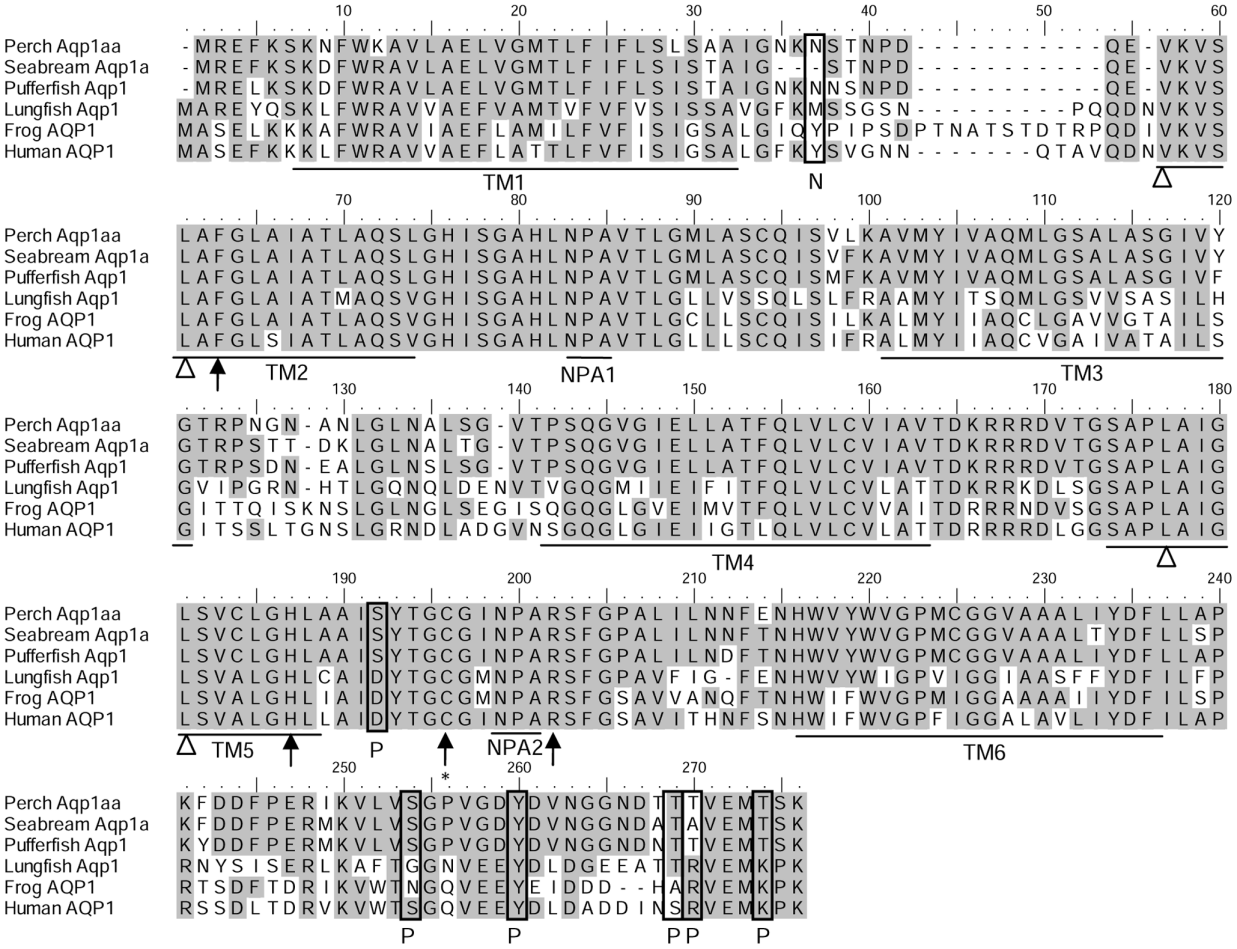
Molecular characterization of aquaporin 1aa (Aqp1aa) from the gills of *Anabas testudineus*. Multiple amino acid alignment of Aqp1aa from the gills of *A. testudineus*, with five other known Aqp1/Aqp1a from *Sparus aurata* (seabream Aqp1a; ABM26907.1), *Takifugu obscurus* (pufferfish Aqp1; ADG86337.1), *Protopterus annectens* (lungfish Aqp1; BAI48049.1), *Xenopus laevis* (frog AQP1; NP_001085391.1), and *Homo sapiens* (human AQP1; CAQ51480.2). Identical amino acids are indicated by shaded residues. Substrate discrimination sites at the aromatic/arginine (ar/R) constriction are indicated with arrows. Central pore-lining residues are indicated with open triangles. The binding site for AQP1-inhibitor HgCl_2_ is indicated by an asterisk. The Asn-Pro-Ala (NPA) motifs are underlined. P denotes phosphorylation sites and N denotes *N*-glycosylation sites. The predicted transmembrane domains (TM) are underlined. The transmembrane domains of Aqp1 of *A. testudineus* were predicted using MEMSATS & MEMSAT-SVA provided by PSIPRED protein structure prediction server.

**Figure 2:**
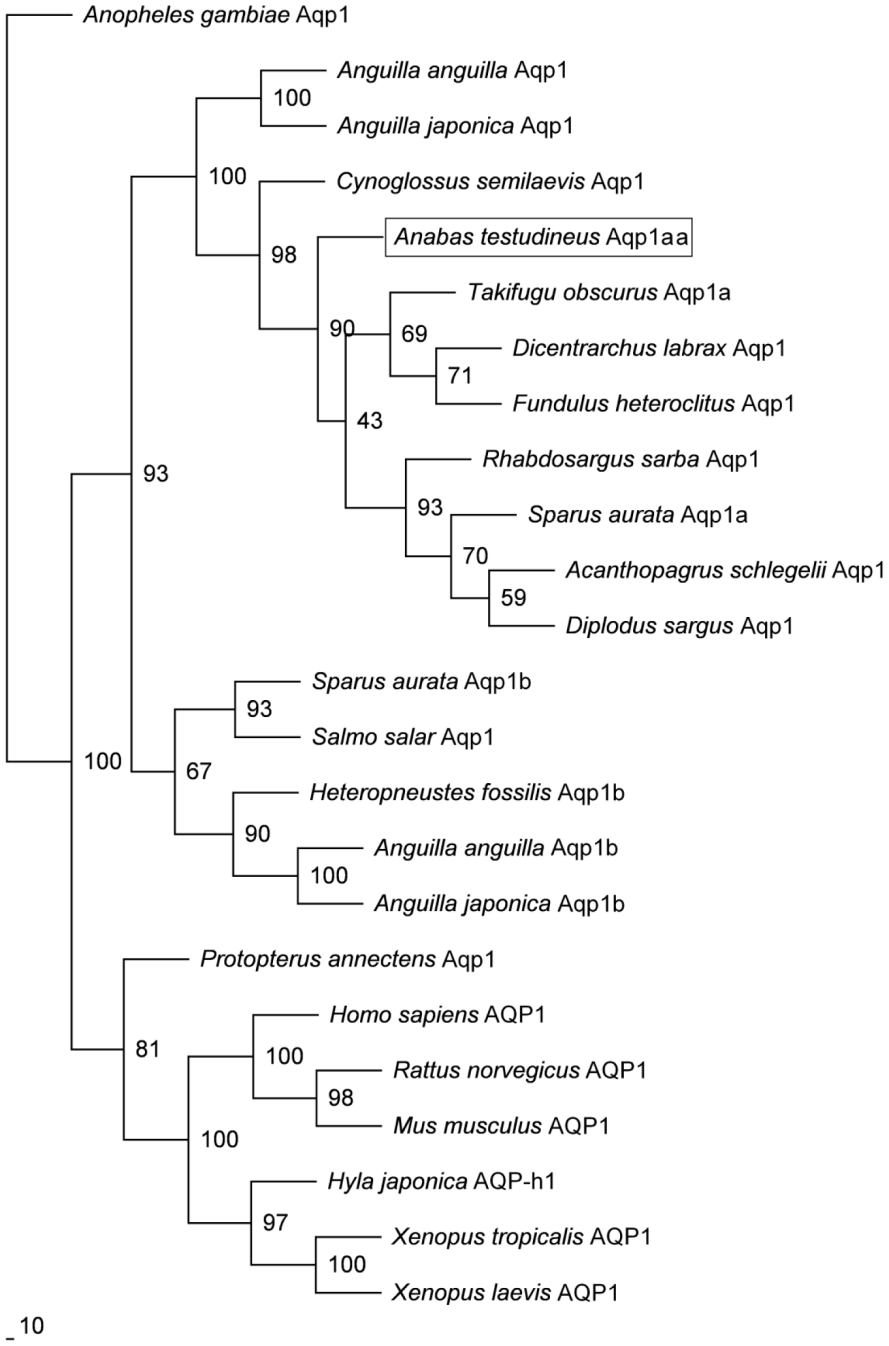
Phylogenetic analysis of aquaporin 1aa (Aqp1aa) of *Anabas testudineus*. The phylogenetic tree illustrates the relationship between Aqp1aa of *A. testudineus* and AQP1/Aqp1 of selected vertebrates.

The complete cDNA coding sequence of *aqp1aa* obtained from the gills of *A. testudineus* consisted of 786 nucleotides (Genbank accession number JX645188), coding for 261 amino acids with an estimated molecular mass of 27.4 kDa (Fig. S1). An alignment of the deduced amino acid sequence of Aqp1aa from *A. testudineus* with those from human, frog and three other fishes (lungfish, pufferfish and seabream) revealed six transmembrane regions, six potential phosphorylation sites and one *N*-glycosylation site (Fig. 1). The substrate discrimination sites at the aromatic/arginine constriction and the asparagine–proline–alanine motifs were conserved. A comparison of *A. testudineus* Aqp1aa with other teleost Aqp sequences reveals that it shares the highest amino acid sequence identity with Aqp1/Aqp1a (67.7–92.3%), followed by Aqp1b (57.5–64.3%; Table 1). This is highly indicative of its identity as Aqp1aa. A phylogenetic analysis further confirms that the Aqp1aa of *A. testudineus* is grouped together with teleost Aqp1/Aqp1a, separated from teleost Aqp1b or lungfish and tetrapod Aqp1 (Fig. 2).

**Table 1:** The percentage sequence identity, arranged in a descending order of similarity, between the deduced amino acid sequence of aquaporin 1aa (Aqp1aa) of *Anabas testudineus* and Aqp sequences of other fish species obtained from GenBank (accession numbers in brackets).

Fish species	Sequence Identity *of Anabas testudineus* Aqp1aa
*Acanthopagrus schlegelii* Aqp1 (ABO38816.1)	92.3%
*Diplodus sargus* Aqp1 (AEU08496.1)	92.3%
*Takifugu obscurus* Aqp1(ADG86337.1)	92.3%
*Sparus aurata* Aqp1a (ABM26907.1)	92.3%
*Dicentrarchus labrax* Aqp1 (ABI95464.2)	91.5%
*Rhabdosargus sarba* Aqp1 (AEG78286.1)	91.1%
*Fundulus heteroclitus* Aqp1 (ACI49538.1)	91.1%
*Cynoglossus semilaevis* Aqp1 (ADG21868.1)	86.9%
*Anguilla anguilla* Aqp1 (CAD92028.1)	82.8%
*Anguilla japonica* Aqp1 (BAC82109.1)	82.1%
*Salmo salar* Aqp1 (NP_001133472.1)	67.7%
*Anguilla anguilla* Aqp1b (ABM26906.1)	64.3%
*Anguilla japonica* Aqp1b (BAK53383.1)	64.0%
*Sparus aurata* Aqp1b (ABM26908.1)	60.1%
*Protopterus annectens* Aqp1 (BAI48049.1)	59.1%
*Heteropneustes fossilis* Aqp1b (ADK87346.1)	57.5%
*Neoceratodus forsteri* Aqp0 (BAH98062.1)	44.9%
*Protopterus annectens* Aqp0 (BAH98061.1)	44.8%
*Danio rerio* Aqp4 (NP_001003749.1)	35.7%
*Danio rerio* Aqp8 (NP_001073651.1)	22.9%
*Anguilla japonica* Aqp8 (BAH89254.1)	22.4%
*Danio rerio* Aqp9 (NP_001171215.1)	22.2%
*Danio rerio* Aqp7 (NP_956204.2)	21.4%
*Danio rerio* Aqp10 (AAH75911.1)	20.8%
*Anoplopoma fimbria* Aqp8 (ACQ57933.1)	20.3%
*Sparus aurata* Aqp8 (ABK20159.1)	20.2%
*Protopterus annectens* Aqp3 (BAI48050.1)	20.0%
*Anoplopoma fimbria* Aqp10 (ACQ58348.1)	20.0%
*Salmo salar* Aqp8 (NP_001167386.1)	19.6%
*Dicentrarchus labrax* Aqp7 (CBN81126.1)	19.4%
*Anguilla japonica* Aqp10 (BAH89255.1)	19.1%
*Anguilla anguilla* Aqp3 (CAC85286.1)	18.9%
*Danio rerio* Aqp3 (AAH44188.1)	18.4%
*Dicentrarchus labrax* Aqp3 (ABG36519.1)	17.0%

### Tissue expression

**Figure 3:**
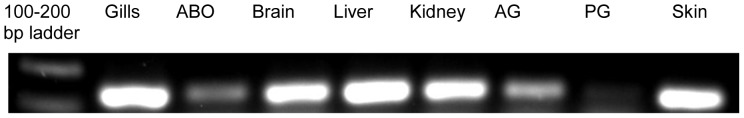
Tissue expression of *aquaporin 1aa* (*aqp1aa*) of *Anabas testudineus* in freshwater. Tissue expression of *aqp1aa* was examined in gills, accessory breathing organs (ABO), brain, liver, kidney, anterior gut (AG), posterior gut (PG) and skin of *A. testudineus* kept in freshwater.

Expression of *aqp1aa* were detected strongly in the gills, brain, liver, kidney and skin, but weakly in the anterior gut, accessory breathing organs and posterior gut (Fig. 3).

### mRNA expression

**Figure 4:**
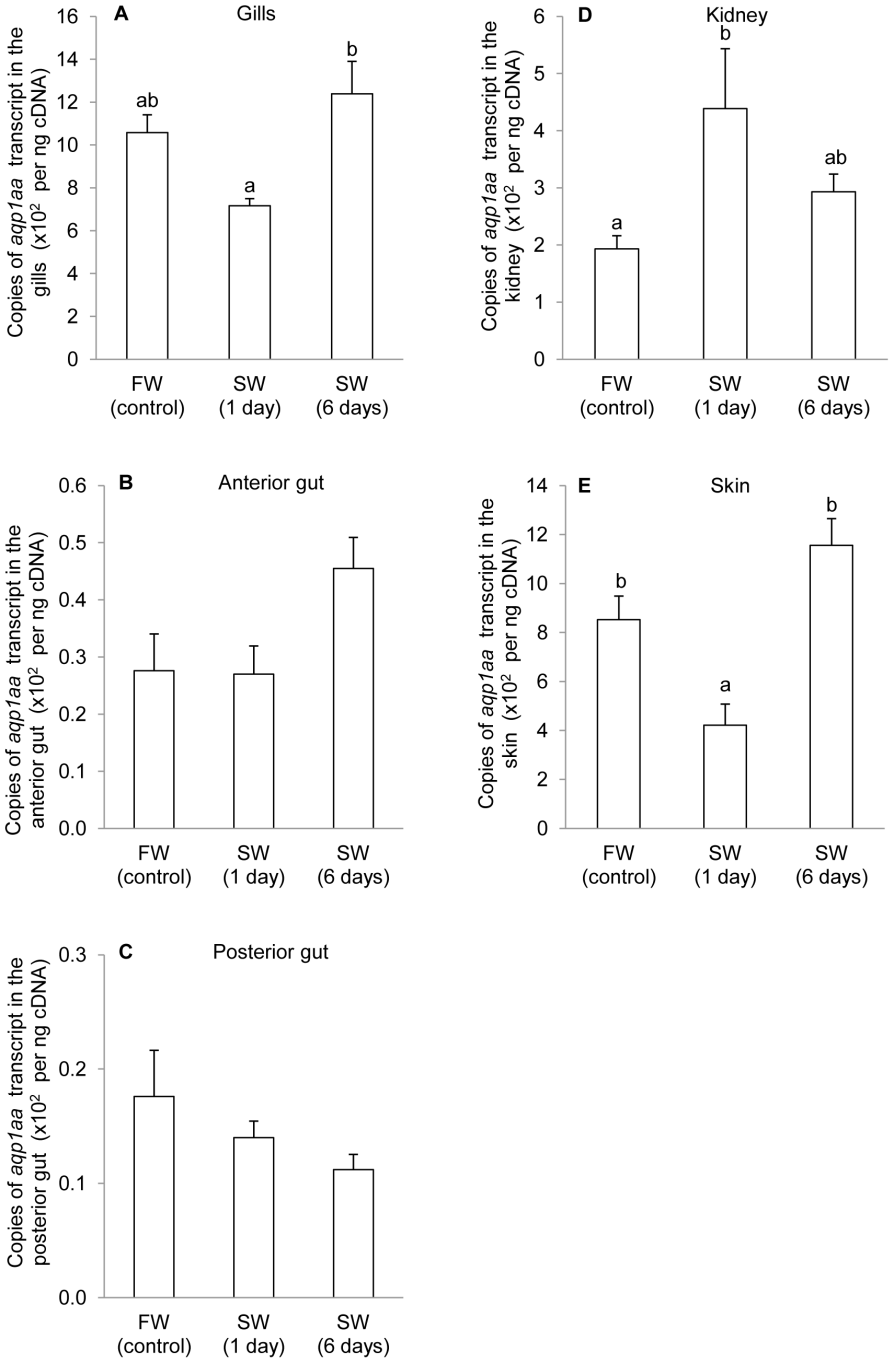
*Aquaporin 1aa* (*aqp1aa*) mRNA expression in *Anabas testudineus* kept in freshwater or seawater. Absolute quantification (×10^2^ copies of transcript per ng cDNA; *N* = 5) of *aqp1aa* mRNA expression from (A) the gills, (B) the anterior gut, (C) the posterior gut, (D) the kidney and (E) the skin of *A. testudineus* kept in freshwater (FW) or exposed to seawater (SW; salinity 30) for 1 or 6 days after a progressive increase in salinity. Results represent means ± S.E.M. Means not sharing the same letter are significantly different (*P*<0.05).

Based on qPCR results, the highest expression of *aqp1aa* mRNA (copies of transcripts per ng cDNA) was detected in gills (∼1000 copies; Fig. 4A), followed by skin (∼800 copies; Fig. 4E) and kidney (∼200 copies; Fig. 4D) of *A. testudineus* in freshwater. In comparison, the mRNA expression of *aqp1aa* in the anterior (∼27 copies; Fig. 4B) and posterior (∼17 copies; Fig. 4C) gut of these fish were very low.

The mRNA expression of *aqp1aa* in the gills (Fig. 4A), anterior gut (Fig. 4B) and posterior gut (Fig. 4C) of *A. testudineus* exposed to seawater for 1 or 6 days after a progressive increase in salinity were comparable to that of the freshwater control. By contrast, 1 day of seawater exposure led to a significant increase and a significant decrease in the mRNA expression of *aqp1aa* in the kidney and skin, respectively; however, such changes were transient and were not observed in fish after 6 days of exposure to seawater.

**Figure 5:**
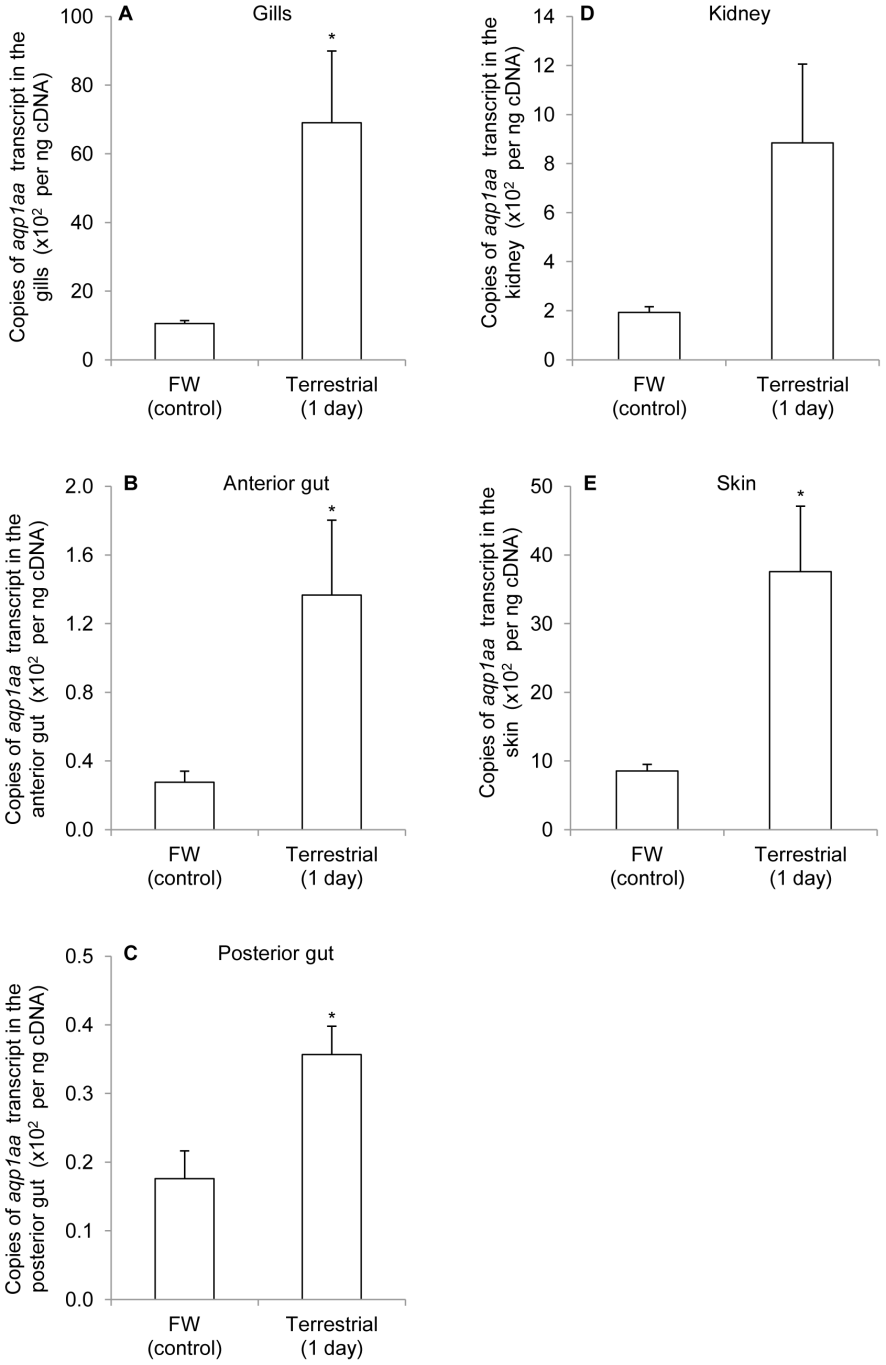
*Aquaporin 1aa* (*aqp1aa*) mRNA expression in *Anabas testudineus* kept in freshwater or exposed to terrestrial conditions. Absolute quantification (×10^2^ copies of transcript per ng cDNA; *N* = 5) of *aqp1aa* mRNA expression from (A) the gills, (B) the anterior gut, (C) the posterior gut, (D) the kidney and (E) the skin of *A. testudineus* kept in freshwater (FW) or exposed to terrestrial conditions for 1 day. Results represent means ± S.E.M. ^*^Significantly different from the FW control (*P*<0.05).

Unlike seawater acclimation, exposure of *A. testudineus* to terrestrial conditions for 1 day resulted in significant increases in mRNA expression of *aqp1aa* in gills (6.53-fold; Fig. 5A), anterior gut (4.95-fold; Fig. 5B), posterior gut (2.03-fold; Fig. 5C) and the skin (4.42-fold; Fig. 5E), but had no significant effect on the kidney (Fig. 5D).

**Figure 6:**
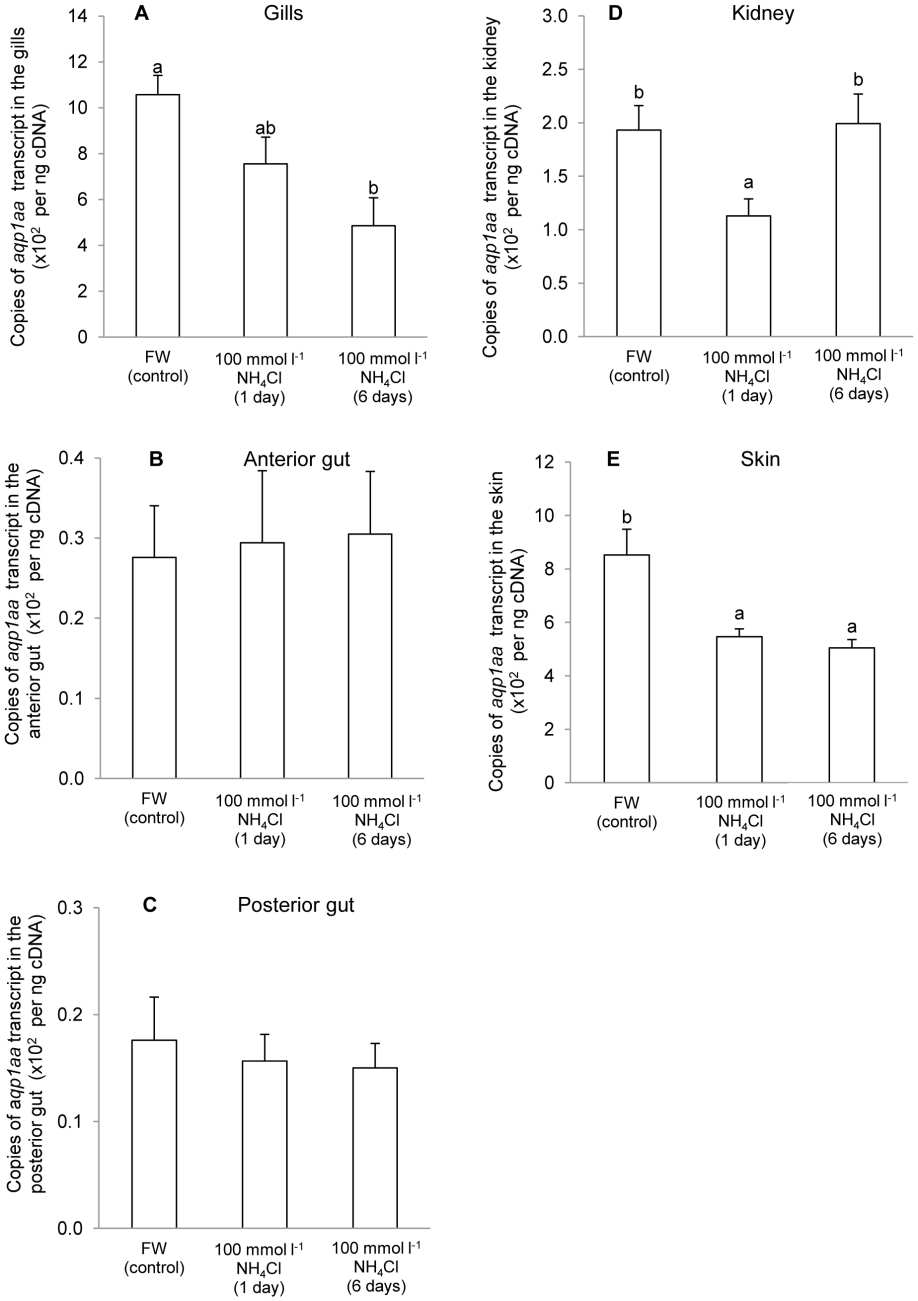
*Aquaporin 1aa* (*aqp1aa*) mRNA expression in *Anabas testudineus* kept in freshwater or exposed to ammonia. Absolute quantification (×10^2^ copies of transcript per ng cDNA; *N* = 5) of *aqp1aa* mRNA expression from (A) the gills, (B) the anterior gut, (C) the posterior gut, (D) the kidney and (E) the skin of *A. testudineus* kept in freshwater (FW) or exposed to 100 mmol l^−1^ NH_4_Cl for 1 or 6 days. Results represent means ± S.E.M. Means not sharing the same letter are significantly different (*P*<0.05).

As for exposure of *A. testudineus* to 100 mmol^−1^ NH_4_Cl in freshwater, results obtained were different from those of terrestrial exposure, with significant decreases in mRNA expression of *aqp1aa* in gills after 6 days (Fig. 6A), kidney after 1 day (Fig. 6D), and skin after 1 or 6 days (Fig. 6E). Exposure to 100 mmol^−1^ NH_4_Cl in freshwater had no significant effects on the mRNA expression of *aqp1aa* in the anterior (Fig. 6B) and posterior (Fig. 6C) gut.

## Discussion

Despite being regarded commonly as a freshwater teleost, *A. testudineus* can acclimate to seawater, survive terrestrial exposure and tolerate high concentrations of environmental ammonia. Since the gills and skin of *A. testudineus* had the highest expression of *aqp1aa*, Aqp1aa could have an important physiological function in these organs. However, the major function of Aqp1aa in *A. testudineus* might not be in osmoregulatory acclimation because of two reasons: (1) seawater acclimation had no significant effects on the mRNA expression of *aqp1aa* in the gills and gut, and (2) the mRNA expression of *aqp1aa* in the gut was extremely low. Terrestrial exposure led to significant increases in the mRNA expression of *aqp1aa* in the gills and skin of *A. testudineus*, but Aqp1aa could not have functioned predominantly in water permeation which would result in deleterious water loss through evaporation. Since it has been established previously that *A. testudineus* utilizes amino acids as energy sources for locomotor activity leading to increased ammonia production while on land [43], it is logical to deduce that increased *aqp1aa* mRNA expression might be necessary to facilitate increased ammonia excretion during emersion. The proposition that Aqp1aa could facilitate ammonia permeation is further supported by the observation that exposure to environmental ammonia led to significant decreases in mRNA expression of *aqp1aa* in the gills and skin, probably to reduce the influx of ammonia during ammonia loading. Hence, our results indicate that Aqp1aa could have a greater physiological role in ammonia excretion than in osmoregulation in *A. testudineus*.

### Molecular characterization of Aqp1aa from the gills of *A. testudineus*: the intrinsic aquapore is permeable to water but not NH_3_

An alignment of the deduced Aqp1aa sequence of *A. testudineus* with those from other species shows highly conserved segments, which include the pore-lining residues of the aquapore, the asparagine–proline–alanine motifs, the AQP1-inhibitor (HgCl_2_) binding site and the outer aromatic/arginine constriction in the aquapore. The substrate discrimination sites of the aromatic/arginine constriction consist of Phe63, His187, Cys189 and Arg202 in *A. testudineus* Aqp1 (corresponding to Phe56, His180, Cys189 and Arg195 in human AQP1). His187 and Arg202 provide a hydrophilic edge with Phe56 [54]. The sulfhydryl group of Cys189 extends into the pore and is the binding site for the AQP1-inhibitor HgCl_2_ [55], [56]. The remaining part of the aquapore contains hydrophobic residues, exposing the main-chain carboxyl oxygens to the pore surface [54]. They act as hydrogen bond acceptor sites to channel small hydrogen bond donor molecules, such as water, through the aquapore.

Beitz et al. [17] analyzed the function of three residues in the aromatic/arginine constriction (Phe56, His180, and Arg195) in rat AQP1. Individual or joint replacement of His180 and Arg195 by alanine and valine, respectively (AQP1-H180A, AQP1-R195V, and AQP1-H180A/R195V), did not affect water permeability, but the double mutant AQP1-H180A/R195V allowed urea to pass through. In line with the predicted solute discrimination by size, replacement of both Phe56 and His180 (AQP1-F56A/H180A) enlarged the maximal diameter of the aromatic/arginine constriction by 3-fold and enabled the passage of glycerol or urea. Beitz et al. [17] showed that NH_3_ could not permeate through the aromatic/arginine constriction of rat AQP1, but it passed through all four AQP1 mutants. Since *A. testudineus* Aqp1aa possesses equivalents of Phe56, His180, and Arg195 in its aromatic/arginine constriction, its intrinsic aquapore probably facilitates water but not NH_3_ movement. However, the possibility of NH_3_ permeation through the central pore of the tetramer cannot be ignored (see below).

### Aqp1aa does not play a major role in osmoregulation in *A. testudineus* during seawater acclimation

To compensate for passive water loss, marine teleosts drink seawater and actively secrete salt via the gills and kidneys. In contrast, freshwater teleosts do not drink (or drink very little) water, but actively absorb salt from the environment through the gills and produce copious hypoosmotic urine to remove excess water via the kidney [57], [58], [59]. Euryhaline teleosts, such as *A. testudineus*, can survive in both freshwater and seawater environments due to their ability to alter osmoregulatory mechanisms upon exposure to media of different salinity.

Fish gills are in direct contact with the surrounding aquatic medium and have a potential risk of large transepithelial water fluxes due to the osmotic gradient [60]. It has been reported that salinity changes would lead to changes in mRNA expression of *aqp1aa/aqp1ab* in the gills of several fish species [29], [30], [31], [32], [60], [61], indicating the involvement of Aqp1aa/Aqp1ab in branchial osmoregulatory acclimation. However, there is no significant difference in the branchial *aqp1aa* mRNA expression between *A. testudineus* exposed to seawater for 1 or 6 days and the freshwater control. From an osmoregulatory perspective, transepithelial water permeability via the branchial epithelium should be kept to a minimum by reducing passive loss of water through a down-regulation of water channels during exposure to hyperosmotic environments. Hence, it can be concluded that, despite having an aquapore that would facilitate water permeation, Aqp1aa does not play a major role in osmoregulation in the gills of *A. testudineus* during seawater acclimation. However, the branchial mRNA expression of *aqp1aa* is the highest among all tissues/organs studied. Therefore, Aqp1aa probably has an important physiological function unrelated to seawater acclimation in the gills of *A. testudineus*.

Marine or seawater acclimatized fish counteract the osmotic loss of water by drinking; and their guts play a crucial osmoregulatory role in water absorption. Several studies have demonstrated the mRNA and/or protein expression of *aqp1aa*/*aqp1ab* in guts of teleost fish [25], [26]. Transcript and protein abundance of *aqp1aa*/*aqp1ab* typically increase towards the distal portions of the gut with the highest level in the posterior region and rectum [22], [23], [31], [60], [62]. The increased Aqp1aa/Aqp1ab expression in the gut would probably contribute to increased water permeability and hence higher rates of intestinal water absorption during seawater acclimation [22], [60], [62]. Indeed, seawater acclimation leads to significant increases in *aqp1aa/aqp1ab* mRNA expression in the guts of several fish species [22], [23], [25], [30], [31], [60], [63]. Furthermore, an injection of cortisol into freshwater eels increases water flux in isolated guts [64] and up-regulates the expression of Aqp1aa throughout the intestines, thereby enhancing intestinal permeability and water absorption [35]. However, the mRNA expression of *aqp1aa* in the anterior and posterior gut of freshwater *A. testudineus* was extremely low and unaffected by seawater acclimation. Thus, it can be concluded that unlike other species of fish, Aqp1aa does not function as a key water channel in water absorption in the gut of *A. testudineus* acclimated to seawater.

In mammalian kidneys, AQP1 is essential for concentrating urine in the thin descending limb of loop of Henle [65]. Kidneys of teleost fish are unable to produce hypertonic urine, but isotonic conditions may be reached in the urine of euryhaline fish during acclimation to hyperosmotic environments. This is largely explained by increased re-absorption of water from the glomerular filtrate in the distal segments of nephrons [66]. In the European eel, Aqp1aa is present within the apical brush border of epithelial cells of a subset of renal tubules, and it seems to be more prominent in the proximal rather than the distal tubules [63], [67]. However, the functional role of Aqp1aa in fish kidney is controversial at present [30], [32], [60], [63], although there are indications that the osmoregulatory role of renal Aqp1aa may be different from that of renal Aqp1ab [26]. For *A. testudineus*, there is a significant increase in the mRNA expression of *aqp1aa* in the kidney after 1 day, but not 6 days, of acclimation to seawater, indicating that Aqp1aa may have at most a transient role in water re-absorption during seawater acclimation.

Today, there is a dearth of knowledge on the response of Aqp1aa to salinity stress in the skin of fish. We report for the first time a decrease in the mRNA expression of *aqp1aa* in the skin of *A. testudineus* after 1 day, but not 6 days, of seawater exposure. The transient nature of this decrease in *aqp1aa* expression in the skin of *A. testudineus* indicates that Aqp1aa might not have a significant role in regulating water loss in a hyperosmotic environment, because the experimental fish were confronted with osmotic water loss not just for 1 day, but throughout the 6 days of acclimation period. Of note, Chang et al. [44] reported that *A. testudineus* reduced ammonia excretion and simultaneously accumulated certain amino acids, presumably for cell volume regulation, during a progressive increase in salinity. However, after long term acclimation to seawater, tissue amino acid concentrations returned to normal, and there is a significant increase in ammonia excretion instead [44]. Since it has been reported previously that AQP1 can also act as an ammonia transporter [15], [18], our results, when taken together with those reported by Chang et al. [44], suggest that Aqp1aa might be involved in increased ammonia excretion in *A. testudineus* during seawater acclimation.

### Up-regulation of mRNA expression of *aqp1aa* and the possible role of Aqp1aa in ammonia excretion in *A. testudineus* during terrestrial exposure

Terrestrial exposure poses a number of challenges to teleosts; the two major problems are (1) desiccation due to water loss, and (2) ammonia intoxication due to inefficient ammonia excretion resulting from a lack of water to flush the branchial epithelium. To the best of our knowledge, there is no information on the effects of terrestrial exposure on the expression of any *aqp* isoform in air-breathing fishes in the literature. Results obtained from this study indicate for the first time that 1 day of terrestrial exposure leads to significant increases in the mRNA expression of *aqp1aa* in several organs, including gills and skin, of *A. testudineus*. To deal with desiccation during terrestrial exposure, it would be essential for *A. testudineus* to reduce water loss through the gills and skin, which have large surface areas. Hence, it is highly unlikely that the increase in expression of *aqp1aa* represents a provision for increased evaporative water loss through the branchial and cutaneous surfaces. This further supports the proposition that Aqp1aa may not function predominantly as a water channel in the gills and skin of *A. testudineus* during osmoregulatory acclimation. AQP1 is known to facilitate CO_2_ permeation [5], but the increased expression of *aqp1aa* in the gills and skin of *A. testudineus* could be unrelated to CO_2_ excretion during emersion. Since *A. testudineus* is an obligatory air-breather and possesses accessory breathing organs for air-breathing, it is unlikely that it would be confronted with problems related to CO_2_ excretion while on land. Rather, our results indicate a possible relationship between increased *aqp1aa* expression and increased ammonia excretion in *A. testudineus* during terrestrial exposure.

Although some aquaporins, such as AQP8 [68], are known to facilitate NH_3_ permeation, whether mammalian AQP1 can enhance ammonia conductance is controversial [15], [16], [18], [54], [69]. The first study on the possible role of AQP1 as an ammonia transporter was performed by Nakhoul et al. [15] who expressed human AQP1 in *Xenopus* oocytes and concluded that it facilitated NH_3_ transport. Subsequently, Holm et al. [16] used *Xenopus* oocytes under open-circuit and voltage-clamped conditions (to exclude NH_4_^+^ and H^+^ transport) to study the effect of several human AQPs on NH_3_ transport by monitoring the rate of acidification of a weakly buffered external medium. They reported that, except for AQP1, expression of AQP3, AQP8, and AQP9 increased acidification, confirming their functional roles in enhancing NH_3_ influx across the cell membrane [16]. Based on a technique similar to that of Holm et al. [16], Musa-Aziz et al. [18] reported recently that AQP1 enhanced NH_3_ influx significantly more than AQP4 and AQP5 in *Xenopus* oocytes, pointing to facilitated transport of NH_3_ by AQP1. While the discrepancies between results of Holm et al. [16] and those of Musa-Aziz et al. [18] could be due to differences in sensitivities of the methods employed in their studies [8], they also point to the possibility that the capacity (or lack thereof) of an AQP channel to conduct ammonia cannot be determined solely by amino acid residues in the aromatic/arginine constriction of the AQP monomer as described by Beitz et al. [17].

Indeed, AQP homologs in yeasts and plants can facilitate ammonia transport, despite having completely dissimilar amino acid residues in the aromatic/arginine constriction [54], [70]. For plant AQP homologs, the tonoplast intrinsic proteins (TIPs) from wheat (TaTIP2;1) and *Arabidopsis* (AtTIP2;1 and AtTIP2;3) can also facilitate NH_3_ transport in addition to being water channels [69], [71]. However, the conduction of water and ammonia through TaTIP2;2 from wheat is differentially affected by inhibitors [72], indicating that NH_3_ permeation may not occur through the monomeric channel pores. Indeed, in the wheat TaTIP2;2, NH_3_ is not transported in file with water, but through a separate pathway, which could be supplied by the fifth central pore in the TaTIP2;2 tetramer conformation [72]. Dynowski et al. [70] conducted molecular simulations on *Arabidopsis thaliana* AtTIP1;2 and AtPIP2;1 (plasma membrane intrinsic protein 2-1) to test the relevance of different selectivity filters, and their results did not support the proposition that an Arg/His pair in the aromatic/arginine selectivity filter region would promote ammonia conductance [54]. They showed that ammonia could cross the membrane via the central pore instead of the aquapores [70]. The fifth central pore can function as a gated channel moderated by cGMP interaction with the cytoplasmic loop D [73]. It consists of mainly hydrophobic amino acids, which provides a path for non-polar molecules [8]. For human AQP1, which is known to facilitate ammonia transport according to Nakhoul et al. [15] and Musa-Aziz et al. [18], amino acid residues with side chains lining the cytoplasmic and periplasmic constriction regions of the central pore include Val50, Leu54, Leu170 and Leu174 (Fig. 1). Since *A. testudineus* Aqp1aa possesses equivalent amino acid residues of Val44, Leu48, Leu162 and Leu166, it is reasonable to suggest that the central pore formed in its tetrameric conformation has the physicochemical potential for ammonia permeation.

Despite the absence of supportive evidence, it has been proposed previously that Aqp would facilitate transepithelial ammonia fluxes in gills of fish [33], [74], [75]. Our results offer for the first time indirect support to the proposition that Aqp1aa could be involved in ammonia excretion through the gills and skin of *A. testudineus*. Since *A. testudineus* utilizes amino acids as energy sources for locomotor activity on land [43], which leads to an increase in ammonia production, it is highly probable that *aqp1aa* expression was up-regulated in gills and skin to facilitate passive ammonia excretion at the initial phase of terrestrial exposure before the buildup of an ammonia electrochemical gradient that requires the participation of active transport mechanisms [45], [46], [47]. A similar functional role in passive NH_3_ permeation has been suggested for AQP8 in the inner mitochondrial membrane of liver cells [76].

In general, the gills are the major site of ammonia excretion in fish, although smaller quantities of ammonia may also be eliminated by the kidney [77]. However, unlike gills and skin, the kidney is not directly exposed to the external environment. More importantly, ammonia excretion through the kidney requires a constant supply of water for urine production. Therefore, the kidney probably plays a minimal role in ammonia excretion terrestrial exposure during which desiccation is a major issue. This could account for the lack of change in *aqp1aa* expression in the kidney of *A. testudineus* after 1 day of exposure to terrestrial conditions.

Of note, while a predisposition based on the type of amino acid residues along the fifth central pore of the Aqp1aa may satisfy certain requirements towards NH_3_ transport, other factors such as the size of, and the orientation of the amino acid residues in, the central pore need to be considered. Additionally, the Aqp1aa tetramer may need to interact with certain protein partners in order for the central pore to act as a NH_3_ channel. Indeed, mammalian AQP1 has been co-immunoprecipitated with transporters such as Na^+^/H^+^ and Cl^−^/HCO_3_^−^ exchangers, and with heterotrimeric complexes of PDZ domain (PDZ is derived from the first three proteins in which these domains were found: PSD-95 which is a 95 kDa protein involved in signaling in the post-synaptic density, Dlg which is the *Drosophila* discs large protein, and ZO1 which is the zonula occludens 1 protein involved in maintaining epithelial cell polarity) proteins [78]. Interactions with other signaling molecules have also been reported, reinforcing the idea that AQP1 may be regulated by multiple levels of signaling cascades, the onset of which depends on the local cytosolic environment and physiological needs [79]. All these could contribute to the differences in physiological functions of Aqp1aa/Aqp1ab in various fish species, and our results indicate that Aqp1aa could have a more predominant physiological role in ammonia excretion than in osmoregulation in *A. testudineus*.

### Down-regulation of mRNA expression of *aqp1aa*, presumably to reduce the influx of exogenous ammonia, during ammonia exposure

Elevated ammonia concentrations in the environment can lead to impaired ammonia excretion and/or a net influx of ammonia from the environment. The end result is an elevation in body ammonia levels, leading to convulsions and death. Most fish species cannot tolerate high environmental ammonia concentrations, but some species, including *A. testudineus*, have high environmental ammonia tolerance [75], [80], [81]. *Anabas testudineus* has the extraordinary ability to actively excrete ammonia (as NH_4_^+^) against an unfavorable blood-to-water P_NH3_ gradient during exposure to environmental ammonia. However, active excretion of ammonia prescribes that the influx or backflux of NH_3_ into the fish must be prevented. The gills are the primary excretory organ where active ammonia excretion occurs in *A. testudineus*. Moreover, its large surface area implies that it would be a major site prone to the influx of external ammonia. One would therefore expect the gills to reduce its permeability through the reduction of Aqp1aa to prevent the entry of ammonia into the fish body, and the same argument can be applied to the skin. Indeed, there are significant decreases in the mRNA expression of *aqp1* in the gills of *A. testudineus* after 6 days of exposure to ammonia, and in the skin of fish exposed to ammonia for 1 or 6 days. Therefore, it is logical to deduce that down-regulation of *aqp1aa* could be an essential mechanism to reduce the net influx of NH_3_in *A. testudineus* during ammonia exposure. These results also corroborate the proposition that increased *aqp1aa* in the gills and skin of *A. testudineus* during terrestrial exposure served to facilitate NH_3_ and not CO_2_ excretion. Besides transcriptional regulation of *aqp1aa* expression, there could be a decrease in the translation of the existing *aqp1aa* transcripts and/or an increase in the removal of Aqp1aa from the cell membranes to prevent NH_3_ influx, the confirmation of which awaits future studies.

### Conclusion

In conclusion, our results indicate that Aqp1aa could have a more prominent physiological role in ammonia excretion than in osmoregulation in *A. testudineus*, which indirectly support previous propositions that AQP1 can act as an ammonia transporter. A molecular characterisation of *A. testudineus* Aqp1aa indicates that its aquapore may not be able to facilitate NH_3_ movement, but highlights the physicochemical potential for ammonia permeation through the central pore of the Aqp1 tetramer. Therefore, efforts should be made in the future to elucidate the functional role of the central pore of the Aqp1aa tetramer in NH_3_ homeostasis in *A. testudineus* and other fish species. Furthermore, future studies should also focus on the functional roles of Aqp3 and Aqp8 in ammonia excretion versus osmoregulation in fish in general.

## Supporting Information

Figure S1 **Nucleotide sequence (GenBank accession number JX645188) and translated amino acid sequence of the full coding region of Aqp1aa from the gills of** ***Anabas testudineus*** **.** The start codon is indicated by the first ATG, while the stop codon is indicated by an asterisk.
(TIF)

## References

[1] King SL, Agre P (2001) Water Channels. Baltimore, MD: John Wiley & Sons, Ltd.

[2] Verbavatz J, Brown D, Sabolić I, Valenti G, Ausiello D, et al (1993) Tetrameric assembly of CHIP28 water channels in liposomes and cell membranes: a freeze-fracture study. J Cell Biol 123: 605–618. 7693713 10.1083/jcb.123.3.605 PMC2200118

[3] Shi L, Skach W, Verkman A (1994) Functional independence of monomeric CHIP28 water channels revealed by expression of wild-type mutant heterodimers. J Biol Chem 269: 10417–10422. 7511600

[4] Gonen T, Walz T (2006) The structure of aquaporins. Q Rev Biophys 39: 361–396. 17156589 10.1017/S0033583506004458

[5] Verkman AS, Mitra AK (2000) Structure and function of aquaporin water channels. Am J Physiol Renal Physiol 278: F13–F28. 10644652 10.1152/ajprenal.2000.278.1.F13

[6] Takata K, Matsuzaki T, Tajika Y (2004) Aquaporins: water channel proteins of the cell membrane. Progr Histochem Cytochem 39: 1–83. 10.1016/j.proghi.2004.03.001 15242101

[7] Preston GM, Agre P (1991) Isolation of the cDNA for erythrocyte integral membrane protein of 28 kilodaltons: member of an ancient channel family. Proc Natl Acad Sci USA 88: 11110–11114. 1722319 10.1073/pnas.88.24.11110 PMC53083

[8] Herrera M, Garvin JL (2011) Aquaporins as gas channels. Eur J Physiol 462: 623–630. 10.1007/s00424-011-1002-x 21809007

[9] Jung JS, Preston GM, Smith BL, Guggino WB, Agre P (1994) Molecular structure of the water channel through aquaporin CHIP. The hourglass model. J Biol Chem 269: 14648–14654. 7514176

[10] Cheng A, van Hoek AN, Yeager M, Verkman AS, Mitra AK (1997) Three-dimensional organization of a human water channel. Nature 387 (6633) 627–630. 9177354 10.1038/42517

[11] Murata K, Mitsuoka K, Hirai T, Walz T, Agre P, et al (2000) Structural determinants of water permeation through aquaporin-1. Nature 407 (6804) 599–605. 11034202 10.1038/35036519

[12] Törnroth-Horsefield S, Hedfalk K, Fischer G, Lindkvist-Petersson K, Neutze R (2010) Structural insights into eukaryotic aquaporin regulation. FEBS Lett 584: 2580–2588. 20416297 10.1016/j.febslet.2010.04.037

[13] Savage DF, Egea PF, Robles-Colmenares Y, O'Connell JD 3rd, Stroud RM (2003) Architecture and Selectivity in Aquaporins: 2.5 Å X-Ray Structure of Aquaporin Z. PLoS Biol 1 (3) e72 doi:10.1371/journal.pbio.0000072. 14691544 10.1371/journal.pbio.0000072 PMC300682

[14] Wang Y, Schulten K, Tajkhorshid E (2005) What makes an aquaporin a glycerol channel: A comparative study of AqpZ and GlpF. Structure 13: 1107–1118. 16084383 10.1016/j.str.2005.05.005

[15] Nakhoul NL, Hering-Smith KS, Abdulnour-Nakhoul SM, Hamm LL (2001) Transport of NH_3_/NH_4_ ^+^ in oocytes expressing aquaporin-1. Am J Physiol Renal Physiol 281: F255–F263. 11457716 10.1152/ajprenal.2001.281.2.F255

[16] Holm LM, Jahn TP, Møller ALB, Schjoerring JK, Ferri D, et al (2005) NH_3_ and NH_4_ ^+^ permeability in aquaporin-expressing *Xenopus* oocytes. Pflugers Arch 450: 415–428. 15988592 10.1007/s00424-005-1399-1

[17] Beitz E, Liu K, Ikeda M, Guggino WB, Agre P, et al (2006) Determinants of AQP6 trafficking to intracellular sites versus the plasma membrane in transfected mammalian cells. Biol Cell 98: 101–109. 15892693 10.1042/BC20050025

[18] Musa-Aziz R, Chen LM, Pelletier MF, Boron WF (2009) Relative CO_2_/NH_3_ selectivities of AQP1, AQP4, AQP5, AmtB, and RhAG. Proc Natl Acad Sci USA 106: 5406–5411. 19273840 10.1073/pnas.0813231106 PMC2664022

[19] Finn RN, Cerdà J (2011) Aquaporin evolution in fishes. Front Physio 2: 44 doi:10.3389/fphys.2011.00044. 10.3389/fphys.2011.00044 PMC3145251 21886623

[20] Cutler CP, Cramb G (2000) Water transport and aquaporin expression in fish. In: Hohmann S and Neilsen SN, editors. Molecular Biology and Physiology of Water and Solute Transport. New York: Kluwer Academic/Plenum Publishers, pp. 433–441.

[21] Martinez AS, Cutler CP, Wilson G, Phillips C, Hazon N, et al (2005) Cloning and expression of three aquaporin homologues from the European eel (*Anguilla anguilla*): effects of seawater acclimation and cortisol treatment on renal expression. Biol Cell 9: 615–627. 10.1042/BC20040111 15850452

[22] Aoki M, Kaneko T, Katoh F, Hasegawa S, Tsutsui N, et al (2003) Intestinal water absorption through aquaporin 1 expressed in the apical membrane of mucosal epithelial cells in seawater-adapted Japanese eel. J Exp Biol 206: 3495–3595. 12939380 10.1242/jeb.00579

[23] Kim YK, Watanabe S, Kaneko T, Do Huh M, Park SI (2010) Expression of aquaporins 3, 8 and 10 in the intestines of freshwater and seawater-acclimated Japanese eels *Anguilla japonica* . Fish Sci 76: 695–702.

[24] Fabra M, Raldúa D, Bozzo MG, Deen PMT, Lubzens E, et al (2006) Yolk proteolysis and aquaporin-1o play essential roles to regulate fish oocyte hydration during meiosis resumption. Dev Biol 295: 250–262. 16643885 10.1016/j.ydbio.2006.03.034

[25] Raldúa D, Otero D, Fabra M, Cerdà J (2008) Differential localization and regulation of two aquaporin-1 homologs in the intestinal epithelia of the marine teleost Sparus aurata. Am J Physiol 294: 993–1003. 10.1152/ajpregu.00695.2007 18171690

[26] Cerdà J, Finn RN (2010) Piscine aquaporins: an overview of recent advances. J Exp Zool A Ecol Genet Physiol 313: 623–650. 20717996 10.1002/jez.634

[27] Tingaud-Sequeira A, Chauvigné F, Fabra M, Lozano J, Raldúa D, et al (2008) Structural and functional divergence of two fish aquaporin-1 water channels following teleost-specific gene duplication. BMC Evol Biol 8: 259–277 doi:10.1186/1471-2148-8-259. 18811940 10.1186/1471-2148-8-259 PMC2564943

[28] Fabra M, Raldúa D, Power DM, Deen PM, Cerdà J (2005) Marine fish egg hydration is aquaporin-mediated. Science 307: 545. 15681377 10.1126/science.1106305

[29] Deane EE, Woo NYS (2006) Tissue distribution, effects of salinity acclimation, and ontogeny of aquaporin 3 in the marine teleost, silver seabream (Sparus sarba). Mar Biotechnol 8: 663–671. 16909214 10.1007/s10126-006-6001-0

[30] Deane EE, Luk JCY, Woo NYS (2011) Aquaporin 1a expression in gill, intestine, and kidney of the euryhaline silver seabream. Front Physiol 2: 39 doi:10.3389/fphys.2011.00039. 21811469 10.3389/fphys.2011.00039 PMC3143732

[31] Giffard-Mena I, Boulo V, Aujoulat F, Fowden H, Castille R, et al (2007) Aquaporin molecular characterization in the sea-bass (*Dicentrarchus labrax*): the effect of salinity on AQP1 and AQP3 expression. Comp Biochem Physiol A 148: 430–444. 10.1016/j.cbpa.2007.06.002 17618150

[32] An KW, Kim NN, Choi CY (2008) Cloning and expression of aquaporin1 and arginine vasotocin receptor mRNA from the black porgy, Acanthopagrus schlegeli: effect of freshwater acclimation. Fish Physiol Biochem 34: 185–194. 18649036 10.1007/s10695-007-9175-0

[33] Brunelli E, Mauceri A, Salvatore F, Giannetto A, Maisano M, et al (2010) Localization of aquaporin 1 and 3 in the gills of the rainbow wrasse *Coris julis* . Acta Histochem 112: 251–258. 19428055 10.1016/j.acthis.2008.11.030

[34] Chaube R, Chauvigné F, Tingaud-Sequeira A, Joy KP, Acharjee A, et al (2011) Molecular and functional characterization of catfish (*Heteropneustes fossilis*) aquaporin-1b: changes in expression during ovarian development and hormone-induced follicular maturation. Gen Comp Endocrinol 170: 162–171. 20937280 10.1016/j.ygcen.2010.10.002

[35] Martinez AS, Wilson G, Phillips C, Cutler CP, Hazon N, et al (2005) Effect of cortisol on aquaporin expression in the oesophagus of the European eel, *Anguilla anguilla* . Annals NY Acad Sci 1040: 395–398. 10.1196/annals.1327.072 15891071

[36] Pethiyagoda R (1991) Freshwater fishes of Sri Lanka. The Wildlife Heritage Trust of Sri Lanka, Colombo. p. 362.

[37] Hughes GM, Munshi JSD (1973) Fine structure of the respiratory organs of the climbing perch, *Anabas testudineus* (Pisces, Anabantidae). J Zool London 170: 201–225.

[38] Munshi JSD, Olson KR, Ojha J, Ghosh TK (1986) Morphology and vascular anatomy of the accessory respiratory organs of the air-breathing climbing perch, *Anabas testudineus* (Bloch). Am J Anat 176: 321–331. 3739954 10.1002/aja.1001760306

[39] Graham JB (1997) Air-breathing Fishes: Evolution, Diversity and Adaptation. Academic Press, London.

[40] Sokheng C, Chhea CK, Viravong S, Bouakhamvongsa K, Suntornratana U, et al.. (1999) Fish migration and spawning habits in the Mekong mainstream: a survey using local knowledge (basin-wide). Assessment of Mekong fisheries: Fish Migration and Spawning and the Impact of Water Management Project (AMFC). AMFP Report 2/99. Vientiane, Lao, P.D.R.

[41] Rahman AKA (1989) Freshwater fishes of Bangladesh Zoological Society of Bangladesh: Department of Zoology, University of Dhaka. p. 364.

[42] Davenport J, Abdul Martin AKM (1990) Terrestrial locomotion in the climbing perch, *Anabas testudineus* (Bloch) (Anbabantidea, Pisces). J Fish Biol 37: 175–184.

[43] Tay YL, Loong AM, Hiong KC, Lee SJ, Tng YYM, et al (2006) Active ammonia transport and excretory nitrogen metabolism in the climbing perch, *Anabas testudineus*, during 4 days of emersion or 10 minutes of forced exercise on land. J Exp Biol 209: 4475–4489. 17079718 10.1242/jeb.02557

[44] Chang EWY, Loong AM, Wong WP, Chew SF, Wilson JM, et al (2007) Changes in tissue free amino acid contents, branchial Na^+^/K^+^-ATPase activity and bimodal breathing pattern in the freshwater climbing perch, *Anabas testudineus* (Bloch), during seawater acclimation. J Exp Zool 307A: 708–723. 10.1002/jez.a.424 17963240

[45] Loong AM, Chew SF, Wong WP, Lam SH, Ip YK (2012) Both seawater acclimation and environmental ammonia exposure lead to increases in mRNA expression and protein abundance of Na^+^:K^+^:2Cl^−^ cotransporter in the gills of the freshwater climbing perch, *Anabas testudineus* . J Comp Physiol B 182: 491–506. 22179410 10.1007/s00360-011-0634-7

[46] Ip YK, Loong AM, Kuah JS, Sim EWL, Chen XL, et al (2012) The roles of three branchial Na^+^/K^+^-ATPase α-subunit isoforms in freshwater adaptation, seawater acclimation and active ammonia excretion in *Anabas testudineus* . Am J Physiol Regul Integr Comp Physiol 303: R112–R125. 22621969 10.1152/ajpregu.00618.2011

[47] Ip YK, Wilson JM, Loong AM, Chen XL, Wong WP, et al (2012) Cystic fibrosis transmembrane conductance regulator-like Cl^−^channel in the gills of the climbing perch, *Anabas testudineus*, is involved in both hypoosmotic regulation during seawater acclimation and active ammonia excretion during ammonia exposure. J Comp Physiol B 182: 793–812. 22526263 10.1007/s00360-012-0664-9

[48] Laiz-Carrión R, Guerreiro PM, Fuentes J, Canario AV, Martín Del Río MP, et al (2005) Branchial osmoregulatory response to salinity in the gilthead sea bream, *Sparus auratus* . J Evol Zool A Comp Exp Biol 303: 563–576. 10.1002/jez.a.183 15945079

[49] Whitehead A, Crawford DL (2005) Variation in tissue-specific gene expression among natural populations. Genome Biol 6: R13. 15693942 10.1186/gb-2005-6-2-r13 PMC551533

[50] Hall TA (1999) BioEdit: a user-friendly biological sequence alignment editor and analysis program for Windows 95/98/NT. Nucleic Acids Symp Ser 41: 95–98.

[51] McGuffin LJ, Bryson K, Jones DT (2000) The PSIPRED protein structure prediction server. Bioinformatics 16: 404–405. 10869041 10.1093/bioinformatics/16.4.404

[52] Felsentein J (1989) PHYLIP–Phylogeny Inference Package (Version 3.2). Cladistics 5: 164–166.

[53] Gerwick L, Corley-Smith G, Bayne CJ (2007) Gene transcript changes in individual rainbow trout livers following an inflammatory stimulus. Fish Shellfish Immunol 22: 157–171. 16762566 10.1016/j.fsi.2006.04.003

[54] Wu B, Beitz E (2007) Aquaporins with selectivity for unconventional permeants. Cell Mol Life Sci 64: 2413–2421. 17571212 10.1007/s00018-007-7163-2 PMC11138416

[55] Preston GM, Jung JS, Guggino WB, Agre P (1993) The mercury-sensitive residue at cysteine 189 in the CHIP28 water channel. J Biol Chem 268: 17–20. 7677994

[56] Zhang R, van-Hoek AN, Biwersi J, Verkman AS (1993) A point mutation at cysteine 189 blocks the water permeability of rat kidney water channel CHIP28k. Biochemistry 32: 2938–2941. 8457558 10.1021/bi00063a002

[57] Evans DH, Piermarini PM, Choe KP (2005) The multifunctional fish gill: Dominant site of gas exchange, osmoregulation, acid-base regulation, and excretion of nitrogenous waste. Physiol Rev 85: 97–177. 15618479 10.1152/physrev.00050.2003

[58] Hwang PP, Lee TH (2007) New insights into fish ion regulation and mitochondrion-rich cells. Comp Biochem Physiol A 148: 479–497. 10.1016/j.cbpa.2007.06.416 17689996

[59] Hwang PP, Lee TH, Lin LY (2011) Ion regulation in fish gills: recent progress in the cellular and molecular mechanisms. Am J Physiol Regul Integr Comp Physiol 301: R28–R47. 21451143 10.1152/ajpregu.00047.2011

[60] Tipsmark CK, Sørensen KJ, Madsen SS (2010) Aquaporin expression dynamics in osmoregulatory tissues of Atlantic salmon during smoltification and seawater acclimation. J Exp Biol 213: 368–379. 20086120 10.1242/jeb.034785

[61] Cutler CP, Cramb G (2002) Branchial expression of an aquaporin 3 (AQP-3) homologue is downregulated in the European eel *Anguilla anguilla* following seawater acclimation. J Exp Biol 205: 2643–2651. 12151370 10.1242/jeb.205.17.2643

[62] Kim YK, Ideuchi H, Watanabe S, Park SI, Huh MD, et al (2008) Rectal water absorption in seawater-adapted Japanese eel *Anguilla japonica* . Comp Biochem Physiol A Mol Integr Physiol 151: 533–541. 18687408 10.1016/j.cbpa.2008.07.016

[63] Martinez AS, Cutler CP, Wilson G, Phillips C, Hazon N, et al (2005) Regulation of expression of two aquaporin homologues in the intestine of the European eel: effects of seawater acclimation and cortisol treatment. Am J Physiol 288: R1733–R1743. 10.1152/ajpregu.00747.2004 15650119

[64] Utida S, Hirano T, Oide H, Ando M, Johnson DW, et al (1972) Hormonal control of the intestine and urinary bladder in teleost osmoregulation. Gen Comp Endocrinol 16: 566–573.

[65] Nielsen S, Frokiaer J, Marples D, Kwon TH, Agre P, et al (2002) Aquaporins in the kidney: from molecules to medicine. Physiol Rev 82: 205–244 doi:10.1152/physrev.00024.2001. 11773613 10.1152/physrev.00024.2001

[66] McDonald MD, Grosell M (2006) Maintaining osmotic balance with an aglomerular kidney. Comp Biochem Physiol 143A: 447–458. 10.1016/j.cbpa.2005.12.029 16483812

[67] Cutler CP, Martinez AS, Cramb G (2007) The role of aquaporin 3 in teleost fish. Comp Biochem Physiol A 148: 82–91. 10.1016/j.cbpa.2006.09.022 17126580

[68] Saparov SM, Liu K, Agre P, Pohl P (2007) Fast and selective ammonia transport by aquaporin-8. J Biol Chem 282: 5296–5301. 17189259 10.1074/jbc.M609343200 PMC3056221

[69] Jahn TP, Moller ALB, Zeuthen T, Holm LM, Klaerke DA, et al (2004) Aquaporin homologues in plants and mammals transport ammonia. FEBS Lett 574: 31–36. 15358535 10.1016/j.febslet.2004.08.004

[70] Dynowski M, Mayer M, Moran O, Ludewig U (2008) Molecular determinants of ammonia and urea conductance in plant aquaporin homologs. FEBS Lett 582: 2458–2462. 18565332 10.1016/j.febslet.2008.06.012

[71] Loque D, Ludewig U, Yuan L, von Wiren N (2005) Tonoplast intrinsic proteins AtTIP2;1 and AtTIP2;3 facilitate NH3 transport into the vacuole. Plant Physiol 137: 671–680. 15665250 10.1104/pp.104.051268 PMC1065367

[72] Bertl A, Kaldenhoff R (2007) Function of a separate NH_3_-pore in Aquaporin TIP2;2 from wheat. FEBS Lett 581: 5413–5417. 17967420 10.1016/j.febslet.2007.10.034

[73] Yu J, Yool AJ, Schulten K, Tajkhorshid E (2006) Mechanism of gating and ion conductivity of a possible tetrameric pore in aquaporin-1. Structure 14: 1411–1423. 16962972 10.1016/j.str.2006.07.006

[74] Wilkie MP (2002) Ammonia excretion and urea handling by fish gills: present understanding and future research challenges. J Exp Zool 293: 284–301. 12115902 10.1002/jez.10123

[75] Ip YK, Chew SF (2010) Ammonia production, excretion, toxicity, and defense in fish: a review. Front Physiol 1: 134 doi:10.3389/fphys.2010.00134. 21423375 10.3389/fphys.2010.00134 PMC3059970

[76] Soria LR, Fanelli E, Altamura N, Svelto M, Marinelli RA, et al (2010) Aquaporin-8-facilitated mitochondrial ammonia transport. Biochem Biophys Res Commun 393: 217–221. 20132793 10.1016/j.bbrc.2010.01.104

[77] Maetz J (1972) Branchial sodium exchange and ammonia excretion in the goldfish *Carrassius auratus*. Effects of ammonia-loading and temperature changes. J Exp Biol 56: 601–620.

[78] Monzani E, Bazzotti R, Perego C, La Porta CAM (2009) AQP1 is not only a water channel: it contributes to cell migration through Lin7/beta-catenin. PLoS ONE 4 (7) e6167. 19584911 10.1371/journal.pone.0006167 PMC2701997

[79] Yool AJ, Weinstein AM (2002) New roles for old holes: ion channel function in aquaporin-1. News Physiol Sci 17: 68–72. 11909995 10.1152/nips.01372.2001

[80] Ip YK, Chew SF, Randall DJ (2001) Ammonia toxicity, tolerance and excretion. In: Wright PA and Anderson PM. Editors. Fish Physiology Vol. 20. San Diego: Academic Press. pp. 109–148.

[81] Chew SF, Wilson JM, Ip YK, Randall DJ (2006) Nitrogen excretion and defense against ammonia toxicity. In: Val AL, Almeida-Val VMF and Randall DJ, editors. Fish Physiology Vol. 21. London: Academic Press. pp. 307–395.

