# Supplementary figures and images for: Molecular Characterization of Branchial aquaporin 1aa and Effects of Seawater Acclimation, Emersion or Ammonia Exposure on Its mRNA Expression in the Gills, Gut, Kidney and Skin of the Freshwater Climbing Perch, Anabas testudineus
Source: PLoS One. 2013 Apr 9;8(4):e61163. doi: 10.1371/journal.pone.0061163 (PMC3621907; doi:10.1371/journal.pone.0061163)

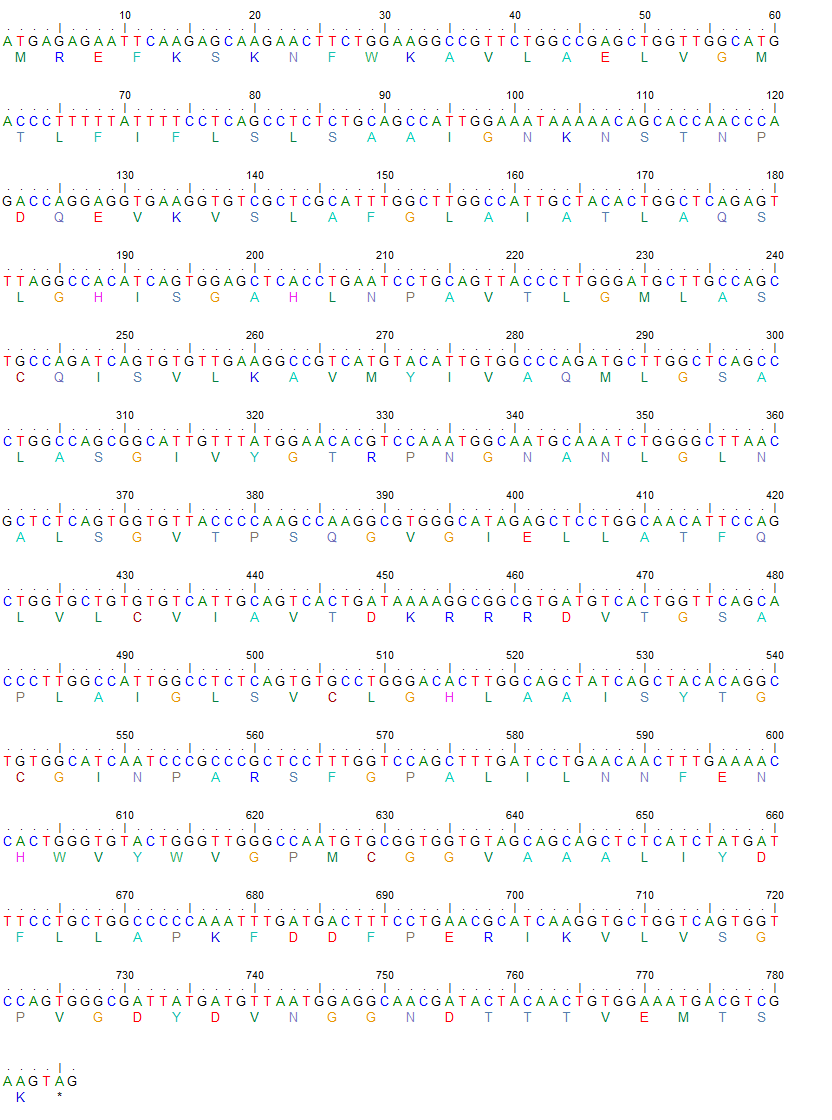

Supplement: Figure S1 — Nucleotide sequence (GenBank accession number JX645188) and translated amino acid sequence of the full coding region of Aqp1aa from the gills of Anabas testudineus . The start codon is indicated by the first ATG, while the stop codon is indicated by an asterisk. (TIF) [file pone.0061163.s001.tif]
